# Comparative Analysis of In Situ Eukaryotic Food Sources in Three Tropical Sea Cucumber Species by Metabarcoding

**DOI:** 10.3390/ani12172303

**Published:** 2022-09-05

**Authors:** Chenghao Jia, Yue Zhang, Qiang Xu, Chunyang Sun, Yanan Wang, Fei Gao

**Affiliations:** 1 State Key Laboratory of Marine Resource Utilization in South China Sea, Hainan University, Haikou 570228, China; 2 College of Ecology and Environment, Hainan University, Haikou 570228, China; 3 College of Marine Sciences, Hainan University, Haikou 570228, China

**Keywords:** sea cucumber, eukaryotic food source, metabarcoding, 18S rRNA

## Abstract

**Simple Summary:**

There are large numbers of sea cucumbers naturally inhabiting tropical seas, which play an important ecological role in the habitat through their biological activity. However, despite the diversity of tropical sea cucumbers, until now there have been few studies on their exact eukaryotic food sources. In the present study, we systemically investigated the eukaryotic food sources of three sea cucumber species. We also study the differences of eukaryotic composition among three sea cucumber species and provide new insight into reasons for the differences. The reported information could be valuable in further biological and ecological studies of these species.

**Abstract:**

In this study, the eukaryotic composition of gut contents in three tropical sea cucumber species, *Stichopus monotuberculatus*, *S. chloronotus* and *Holothuria atra* were surveyed and compared by metabarcoding analysis based on 18S rRNA gene V4 region. The sequences were assigned to 21.80 ± 1.07, 22.60 ± 0.68 and 22.40 ± 0.25 different phyla from the gut contents of *S. monotuberculatus*, *S. chloronotus* and *H. atra,* respectively, and those in sediment samples were assigned to 21.00 ± 1.67 phyla. The results of α-diversity showed that surface sediments had a greater eukaryotic diversity than gut contents, yet the guts of sea cucumbers had an enrichment effect on some microorganisms, including Diatomea and Apicomplex. A comparison of the gut eukaryotic community among the three species suggested that the feeding preference was different: *S. monotuberculatus* fed mainly on Diatomea and Arthropoda, and the other two species had higher Apicomplexa concentrations, which may be due to differences in the morphology of the tentacles and habitat preferences. Moreover, obvious different eukaryotic community composition in the gut contents of the three sea cucumber species and the surrounding sediments also might result from the animals’ selective feeding for sediment patches. The current study filled in gaps about feeding mechanisms of tropical sea cucumbers and provided a basis for further exploring the mechanism about selective feeding and sea cucumber–sediment interaction in the future.

## 1. Introduction

Sea cucumbers (Echinodermata: Holothuroidea) are large and abundant members of marine benthic communities, occurring in a vast array of habitats from wave-exposed zones on coral reefs to deep soft-bottom cold-temperate habitats [1]. Most sea cucumbers are deposit-feeders feeding on organic detritus mixed with silt and sand in surface sediments. The burying behavior by certain species disturbs the upper and sub-surface sediment layers [2], and this bioturbation to sediments has numerous effects on biogeochemical cycles’ [3,4,5]. For most deposit-feeding holothurians, their feeding activity could change the organic load, redistributing surface sediments [5,6,7]. Digestion by holothuroids enhances conversion of organic matter into inorganic forms, which in turn enhances the productivity of benthic biota by excreting inorganic nitrogen and phosphorus, thus recycling organic matter [8,9]. In coral reef environments, where inorganic nutrients are sparse, organic matter recycling has been considered to be one of the main ecosystem functions of holothuroids [8].

Exploring the food source of holothurians is critical for understanding the nutrition recycling in benthic recycling system. Yingst (1976) found that the sea cucumber *Parastichopus pavimensis* preferred to feed on bacteria and fungi rather than plant detritus in a laboratory experiment with 14C labelled food [10]. Moriarty (1982) found *Holothuria atra* and *Stichopus chloronotus* on the Great Barrier Reef could selectively eat bacteria and nitrogenous components of the organic matter through comparing organic carbon and nitrogen and bacterial biomass in the sediments and gut contents [11]. Holothurian *Apostichopus japonicus* is one of the most commercially important temperate species, and its diet has been investigated using various methods including traditional visual methods [12], fatty acid biomarkers [13,14], stable isotope analysis [15] and so on. However, all of the above methods cannot assign a precise taxonomic identity to diets of sea cucumbers.

With the development of molecular biology, metabarcoding techniques have been applied to study the diets of animals, whose diet would be otherwise difficult to determine, and show increased accuracy and sensitivity over traditional methods [16,17,18,19,20]. Previous studies showed holothurians mostly digest bacteria, cyanobacteria, decaying plant (e.g., seagrass and algae) matter, some diatoms, foraminiferans, fungi and other organic matter that constitute detritus [10,11,21,22], so food sources of holothurians fall into two categories, that is, prokaryotes and eukaryotes. In terms of prokaryotes, bacterial community composition in gut contents in several species of holothurians have been studied by 16S rRNA gene sequence analysis [23,24,25,26]. These studies showed that the main prokaryotes in the gut contents of sea cucumbers included Proteobacteria, Actinomycetes, Bacteroidetes, Acidobacteria, Actinobacteria, Verrucomicrobia and some complex flora, and different sea cucumbers may feed on different bacteria. However, as for holothurians’ eukaryotic food sources, so far, only a few studies have been conducted with metabarcoding techniques. The eukaryotic organism composition in gut contents of the sea cucumber *A. japonicus* were investigated by 18S rRNA gene high-throughput sequencing, and 24 to 28 phyla of eukaryotic organisms were identified as food sources [27]. Yamazaki et al. (2020) determined the eukaryotic communities in the feces of the sea cucumber *A. japonicus* through 16S rRNA gene sequencing and considered 12 families including Chaetocerotaceae and Laminariaceae be the main diets. To date, this technology has not been used to study the eukaryotic food sources of other holothurians [28].

*S. monotuberculatus*, *S. chloronotus* and *H. atra* are all common macrobenthos belonging to Holothuroidea in tropical coral reefs. In this study, we compared α-diversity estimates, relative abundances of OTUs and overall eukaryotic composition between the gut contents of the three species of holothurians and the surrounding sediments by metabarcoding. The objectives of this study were to characterize the eukaryotic food sources and analyze the feeding strategy of the three species of commercially exploited tropical sea cucumbers.

## 2. Materials and Methods

### 2.1. Sample Collection

**Figure 1:**
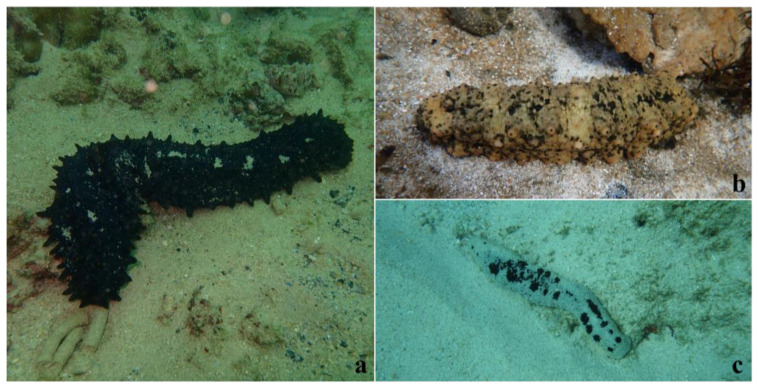
Samples and surrounding environment taken in the process of sample collection from Wuzhizhou island: (**a**) *S. chloronotus*; (**b**) *S. monotuberculatus*; (**c**) *H. atra*.

There are no ethical implications for this study. All sea cucumbers were randomly collected from the sea area around Wuzhizhou Island in Haitang Bay of Hainan Province, China (Figure 1). According to our previous observation, *S. monotuberculatus* usually lived under coral reefs or rocks, yet *S. chloronotus* and *H. atra* often lived in the surrounding sandy bottom area. During the sampling process, according to the principle of community ecotone, we assumed that the sediments at the junction of the two habitats had the common characteristics of the sediments of the two habitats, so the sediments we collected were located at the junction of the two habitats, and three sea cucumber species were also randomly collected near the junction. As these sea cucumbers lived in a natural habitat, they fed only on natural diets. *S. monotuberculatus* (Sm), *S. chloronotus* (Sc), and *H. atra* (Ha) individuals were collected in June, 2020. Ambient surface sediments (Sd, 0–1 cm) were taken separately from 5 locations around the sampled sea cucumbers using 50 mL syringe samplers [25]. Five biological replicates were performed for each group of samples (*n* = 5). Upon collection, sea cucumbers were immediately transported to the laboratory on Wuzhizhou island. 

Each sample was dissected aseptically using alcohol-sterilized dissecting tools. Only the contents in the anterior part of the foregut were taken as the gut contents samples. Gut contents and marine sediments samples were preserved at −80 °C for later analysis.

### 2.2. DNA Extraction and PCR Amplification

According to the manufactuer’s protocol, DNA was extracted from gut contents samples and sediment samples using the Soil DNA Kit (Omega Biotech, USA) in the laboratory, and the purity and concentration of DNA were detected by agarose gel electrophoresis. An appropriate amount of DNA was taken and diluted to 1 ng/μL with sterile water. Based on the selection of sequencing regions, the universal primer set, 528F (5′-GCGGTAATTCAGCTCAA-3’) and 706R (5′-AATCC RAGAATTTCACCTCT-3′) [29], was used for amplification of the V4 region of the 18S rRNA gene from all samples. The composition of the reaction mixture referenced Gao et al. (2014a) [25].

### 2.3. High-Throughput Sequencing

According to the manufacturer’s protocol, sequencing libraries were generated using the TruSeq^®^ DNA PCR-Free Sample Preparation Kit (Illumina, San Diego, CA, USA). The library concentration was assessed on the Qubit@ 2.0 Fluorometer (Thermo Scientific, Carlsbad, CA, USA) system. Finally, the library was sequenced on a Sequencing performed by the NovaSeq6000 platform and 250 bp paired-end reads. All data were sequenced by Novogene (Tianjin, China).

### 2.4. Data Analysis

Quality filtering on the raw reads was performed under specific filtering conditions to obtain the high-quality clean reads according to the Cutadapt quality controlled process [30] (V1.9.1, http://cutadapt.readthedocs.io/en/stable/ (accessed on 3 September 2020)). FLASH (V1.2.7, http://ccb.jhu.edu/software/FLASH/ (accessed on 3 September 2020)) [31] was used to merge read pairs of each sample to raw reads. We used QIIME (V1.9.1, http://qiime.org/scripts/split_libraries_fastq.html (accessed on 3 September 2020)) [32] to finish the reads quality control process and filter out the reads which continuous high quality base length is less than 75% of the reads’ length. An algorithm [33] was used to detect chimera sequences (http://www.drive5.com/usearch/manual/uchime_algo.html (accessed on 3 September 2020)), and then the chimera sequences were removed. Finally, the clean reads were obtained. 

Sequence analysis was performed by Uparse software [34] (v7.0.1001, http://drive5.com/uparse/ (accessed on 8 September 2020)). Then a representative sequence for each OTU was screened for further annotation. Sequences were classified with the RDP Classifier 2.2 (http://sourceforge.net/projects/rdp-classifier/ (accessed on 8 September 2020)) [35] method and Silva132 database (http://www.arb-silva.de/ (accessed on 8 September 2020)) (threshold: 0.6–1) [36]. After examination of the alpha rarefaction curves (Figure S1), samples were rarified to 54,761 sequences per sample.

To calculate α-diversity, we rarified the OTU table and calculated these metrics: Observed-species, Chao-the Chao1 estimator (http://scikit-bio.org/docs/latest/generated/skbio.diversity.alpha.chao1.html#skbio.diversity.alpha.chao1 (accessed on 10 September 2020)), Simpson-the Simpson index (http://scikit-bio.org/docs/latest/generated/skbio.diversity.alpha.simpson.html#skbio.diversity.alpha.simpson (accessed on 10 September 2020)), Shannon-the Shannon index (http://scikit-bio.org/docs/latest/generated/skbio.diversity.alpha.shannon.html#skbio.diversity.alpha.shannon (accessed on 10 September 2020)) and ACE-the ACE estimator (http://scikit-bio.org/docs/latest/generated/skbio.diversity.alpha.ace.html#skbio.diversity.alpha.ace (accessed on 10 September 2020)). All the indices in our samples were calculated with QIIME (Version1.7.0) and displayed with R software (Version 2.15.3, including packages *ggplot2*, *ggpubr*, *ggsignif*, *vegan*, *ggprism*, *picante*, *dplyr*, *RColorRrewer*).

To find the differences between groups at the phylum level, independent samples T-test was performed by R software (Version 2.15.3). The visualization was completed through Prism 9 software, and the relative abundance of species with significant differences among groups was compared. Finally, in order to avoid the occurrence of “Type I error”, we corrected the *p*-value to q-value by the Benjamini and Hochberg (BH) method as follows: (1) the *p*-values of each gene were ranked from the smallest to the largest; (2) the largest *p*-value remains as it is; (3) the second largest *p*-value is multiplied by the total number of genes in a gene list divided by its rank. If less than 0.05-it is significant: q-value = *p*-value * (n/n − 1); and (4) The third *p*-value is multiplied as in step 3: q-value = *p*-value * (n/n − 2); (5) and so on [37,38].

To analyze differences between sample groups, we used the algorithm based on weighted-unifrac distance for nonmetric multidimensional scaling (NMDS) and principal coordinate analysis (PCoA), and the principal coordinate combination with the largest contribution rate was selected for drawing display. R software (Version 2.15.3) was used to draw PCoA and NMDS plots. WGCNA (*weighted gene co-expression network analysis*), *stats* and *ggplot2* packages of R software were used for PCoA analysis, and *vegan* package of R software was used for NMDS analysis. Moreover, clustering among different groups was built by the unweighted pair group method with arithmetic mean (UPGMA), which could interpret the distance matrixing to abundance of OTUs [39].

All the above analysis methods used default parameters for calculation except for the specific parameters mentioned.

## 3. Results

After quality filtering and removal of chimeras, the effective read numbers for each sample ranged from 60,292 to 69,795 with average length 307 bp clustered into OTUs (similarity 97%).

### 3.1. Richness and Diversity Analysis of Sample Communities

**Figure 2:**
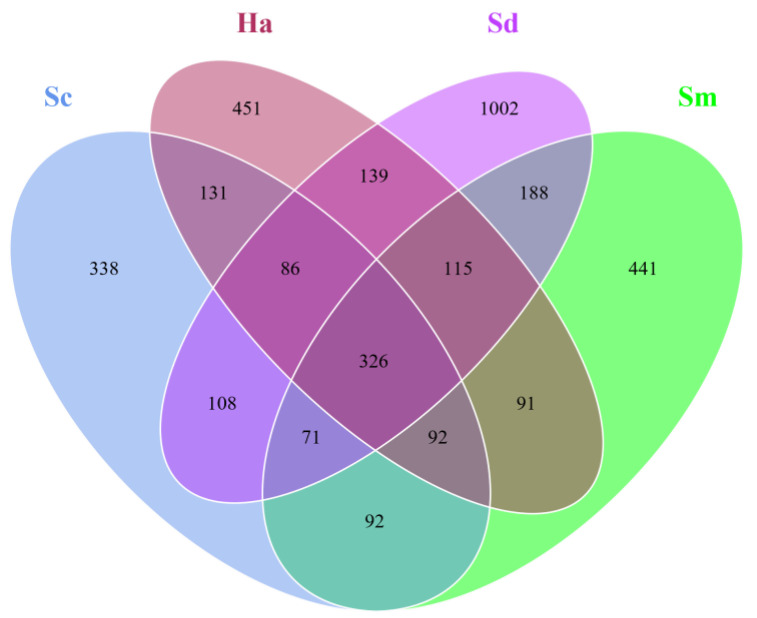
Venn diagram of core OTUs among the gut contents of *S. monotuberculatus* (Sm), *S. chloronotus* (Sc), *H. atra* (Ha) and the surrounding sediments (Sd).

OTUs identified as the host sea cucumber species were first removed, and a total of 3679 OTUs were finally obtained from all the samples. The gut content samples from sea cucumbers *S. monotuberculatus*, *S. chloronotus* and *H. atra* contained 1416, 1244 and 1431 OTUs respectively, and the five sediment samples contained 2035 OTUs (Figure 2). Of these OTUs, 441, 338, 451 and 1002 OTUs were uniquely detected in Sm, Sc, Ha and Sd samples, respectively. Only 326 OTUs (8.86%) were shared by all the gut contents and surrounding sediment samples; 418 OTUs (11.36%) were shared by the gut contents of the three species of sea cucumbers.

**Figure 3:**
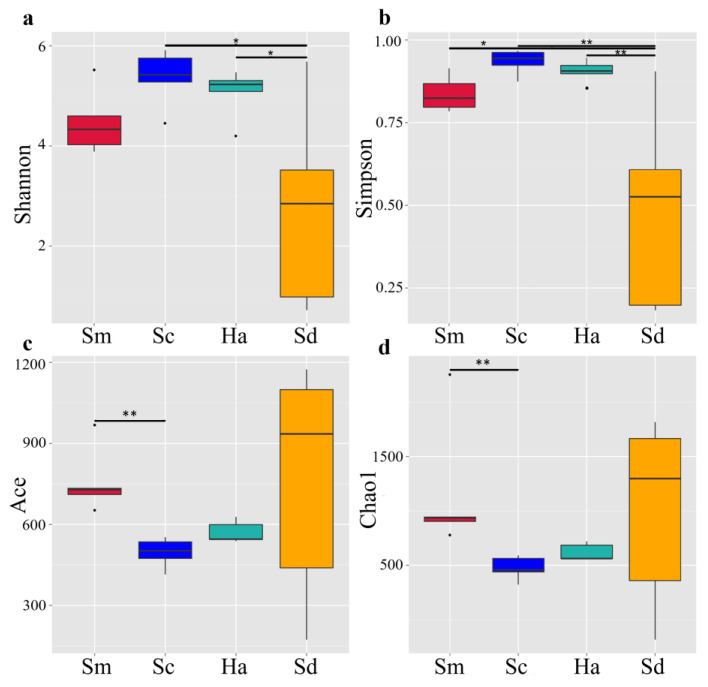
The α-diversity of eukaryotic organism communities in the foregut of *S. monotuberculatus* (Sm), *S. chloronotus* (Sc), *H. atra* (Ha) and the surrounding sediments (Sd): (**a**) Shannon index; (**b**) Simpson index; (**c**) ACE estimator; (**d**) Chao1 estimator. The differences between groups are represented by the differences in the α-diversity index, * *p* < 0.05; ** *p* < 0.01.

Four indices (Shannon, Simpson, Chao1, ACE) were used to assess α-diversity (Figure 3). Indices Shannon and Simpson were applied to evaluate species diversity, and indices Chao1 and ACE were applied to evaluate species richness. The two indices of each part were double-checked to make our results more reliable. The method has been successfully performed in community diversity research [40,41]. Among the α-diversity metrics, the ACE (Abundance-based Coverage Estimator) index in sediments (764.36 ± 195.30) was higher than that in the gut contents of *S. monotuberculatus* (758.95 ± 54.26), *S. chloronotus* (496.10 ± 24.32) and *H. atra* (571.00 ± 17.83), and the chao1 index in sediments (745.56 ± 193.50) was only lower than that in the gut contents of *S. monotuberculatus* (831.10 ± 136.91, Figure 3b). Moreover, the Shannon diversity index and the Simpson diversity index in the sediments both were significantly lower than the indices in the gut contents samples (Figure 3c,d).

### 3.2. Eukaryotic Composition Analysis in Gut Contents and Sediments

**Figure 4:**
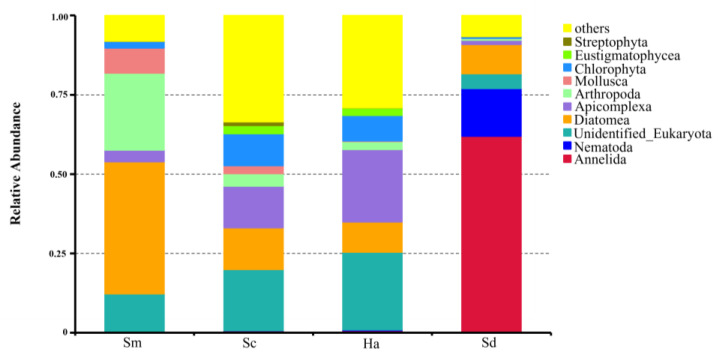
Relative abundance of the 10 most abundant phyla of *S. monotuberculatus* (Sm), *S. chloronotus* (Sc), *H. atra* (Ha) and the surrounding sediments (Sd). Others indicate all reads except the top 10 phyla.

An average of 21.80 ± 1.07, 22.60 ± 0.68 and 22.40 ± 0.25 phyla were identified from the gut contents of *S. monotuberculatus*, *S. chloronotus* and *H. atra,* respectively, and there were 21.00 ± 1.67 phyla in the sediment samples. The 10 most abundant phyla, accounting for 48.13–90.47% of the total reads are shown in Figure 4.

For *S. monotuberculatus*, Diatomea (41.61 ± 4.97%) and Arthropoda (24.27 ± 5.45%) were the predominant phyla, and the relative abundance of Mollusca, Apicomplexa and Chlorophyta were also more than 1%. As for holothurian *S. chloronotus*, there were nine phyla whose relative contents exceeded 1%. Among them, Diatomea (13.15 ± 1.48%), Apicomplexa (13.13 ± 4.77%) and Chlorophyta (10.05 ± 3.57%) were relatively high. In regard to sea cucumber *H. atra*, Apicomplexa (22.85 ± 7.33%) was the predominant phylum, and the contents of Diatomea, Chlorophyta, Arthropoda and Eustigmatophyceae were also over 1%. In the sediment samples, Annelida (61.88 ± 16.59%) and Nematoda (15.04 ± 14.59%) were the predominant eukaryotic organisms, and the contents of Diatomea and Platyhelminthes were also relatively high with the relative abundance over 1%. 

**Figure 5:**
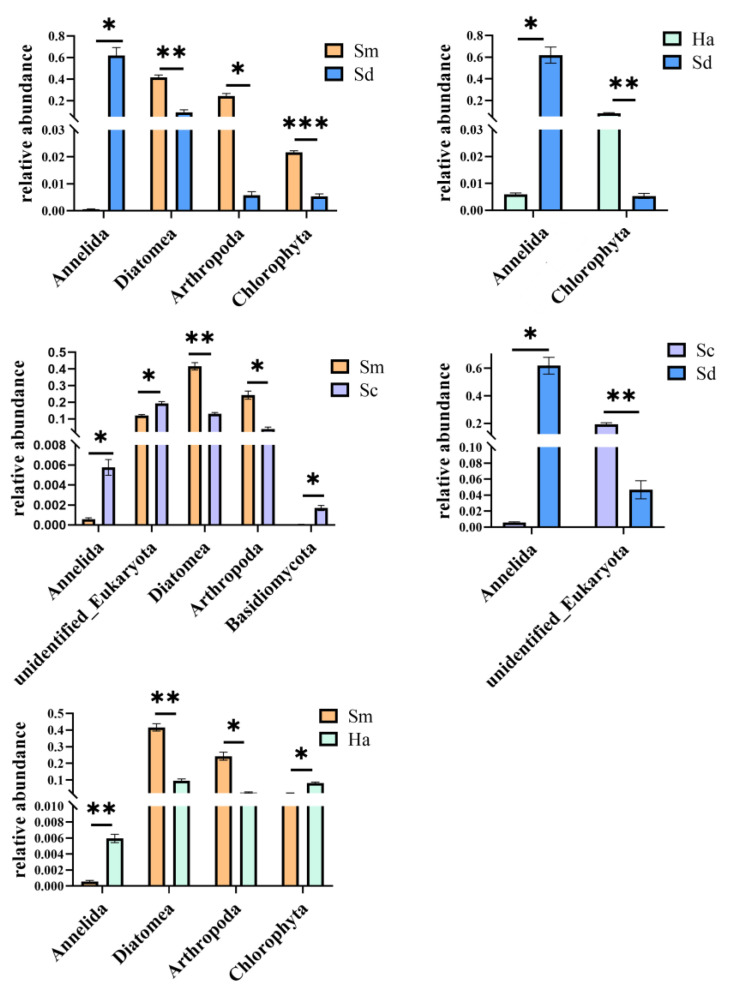
Identified differentially abundant taxa between samples by *t*-test (*p* < 0.05): * *p* < 0.05; ** *p* < 0.01; *** *p* < 0.001.

In order to find the differences between groups at level of phylum, T-test was performed to determine the species with significant differences (*p* < 0.05, q < 0.05). The results of all comparisons are shown in Figure 5. The abundance of Annelida in sediments was significantly higher than that in gut contents of all the species of sea cucumbers (*p* < 0.05, q < 0.05). In *S. monotuberculatus*, the read contents from Diatomea, Arthropoda and Chlorophyta were significantly higher than those in ambient sediment (*p* < 0.05, q < 0.05). In *H. atra*, the contents of Chlorophyta were significantly higher than those in sediments (*p* < 0.05, q < 0.05).

There were no significant differences between *S. chloronotus* and *H. atra* at the phylum level (*p* > 0.05, q > 0.05). In contrast to this, the relative abundance of Diatomea and Arthropoda were significantly higher in *S. monotuberculatus* than in *S. chloronotus* (*p* < 0.05, q < 0.05), but the relative abundance of Annelida and Cnidaria were significantly lower in *S. monotuberculatus* than in *S. chloronotus* (*p* < 0.05, q < 0.05). While comparing the eukaryotic organism contents between *S. monotuberculatus* and *H. atra*, the contents of Diatomea and Arthropoda in *S. monotuberculatus* were significantly higher than those in *H. atra*, and the contents of Annelida and Chlorophyta in *S. monotuberculatus* were significantly lower than those in *H. atra* (*p* < 0.05, q < 0.05).

Table 1 lists the OTUs whose abundance was greater than 10% in any single gut content sample. Moreover, OTUs with abundance above 1% but below 10% are shown in Table S1. An OTU (OTU_1) identified as *Psammodictyon constrictum*, a species of diatom, existed in all five gut content samples of *S. monotuberculatus*, indicating it was a major component of *S. monotuberculatus* gut contents. OTU 6 and OTU 185, both identified as *Loxocorniculum mutsuense*, were found in high abundance in three gut content samples of *S. monotuberculatus*. OTU_7 (identified as a species of *Lankesteria*), OTU_27 (a species of gastropods), OTU_4 (an unidentified eukaryote), OTU_15 (identified as *Acartia pacifica*), OTU_17 (an unidentified eukaryote) and OTU_12 (a species belonging to Chlorophyta) were found in high abundance in the gut content of sea cucumber *S. chloronotus*. For the sea cucumber *H. atra*, OTU_7 (an unidentified Apicomplexa), OTU_4 (an unidentified eukaryote), OTU_8 (an unidentified eukaryote), OTU_10 (*Chromerida* sp.), OTU_3219 (*Chromerida* sp.) and OTU_11 (an unidentified Apicomplexa) had comparatively high abundance.

### 3.3. Relationships of Eukaryotic Communities among the Gut and Sediment Samples

**Figure 6:**
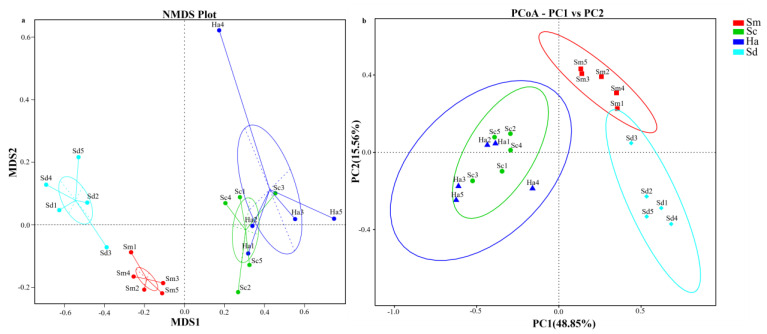
(**a**) Nonmetric multidimensional scaling (NMDS) and (**b**) principal co-ordinates analysis (PCoA) plot based on weighted-unifrac distance showing the relatedness of the eukaryotic composition between the different samples. The explanations of abbreviation in figure: Sm (*S. monotuberculatus*); Sc (*S. chloronotus*); Ha (*H. atra*); Sd (sediment).

**Figure 7:**
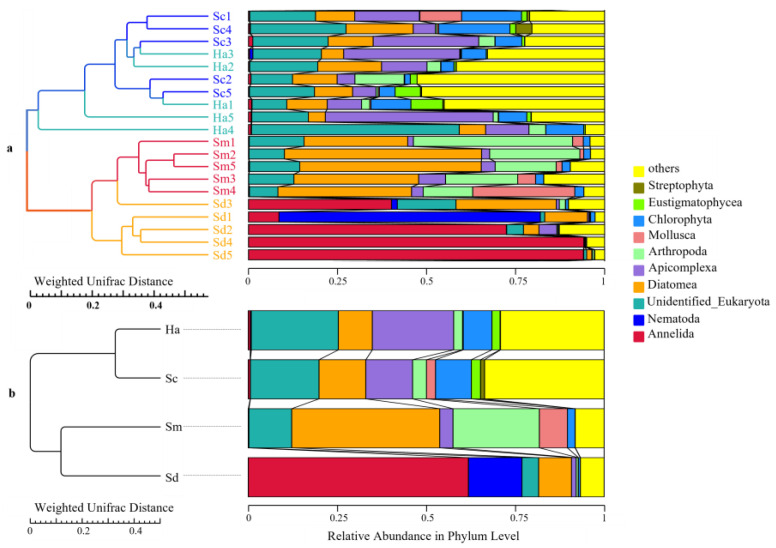
UPGMA clustering tree at the level of phylum based on weighted-unifrac distance showing the similarity of eukaryotic composition among the different samples (**a**) and groups (**b**). The explanations of abbreviation in figure: Sm (*S. monotuberculatus*); Sc (*S. chloronotus*); Ha (*H. atra*); Sd (sediment).

NMDS and PCoA analysis were performed to assess the similarity of the eukaryotic composition among different samples (Figure 6). The analyses indicated that the samples from sediments and guts of the three species of sea cucumbers were clustered separately into three groups: all the sediment samples, gut content samples of *S. monotuberculatus*, and all the gut content samples from *S. chloronotus* and *H. atra*. UPGMA clustering tree at the level of phylum (Figure 7) was in agreement with the results of the NMDS analysis. The results indicated that the gut contents of the three species of sea cucumbers have different characteristic eukaryotic composition with surrounding sediments, while sea cucumber *S. chloronotus* has similar food sources with *H. atra*. Moreover, all analyses showed that compared with *S. chloronotus* and *H. atra*, the intestinal eukaryotic microbial composition of *S. monotuberculatus* was much closer to that of sediment.

## 4. Discussion

Sea cucumbers are prominent members of benthic communities distributed in oceans all around the world. Most of the studied species are deposit feeders, gathering organic detritus and sediments from the seafloor [42,43,44,45]. As typical deposit-feeding species, *S. monotuberculatus*, *S. chloronotus* and *H. atra* all have a complex diet derived from different sources. Although three different species of holothurians all had a wide range of diets, there were still differences in specific ingredients. In this study, we detected differences in the eukaryotic community composition in digestive tracts of the three commercially exploited tropical holothurians and in their surrounding sediments by the 18S rRNA gene high-throughput sequencing method to determine their food sources and analyze their different feeding strategy.

### 4.1. Dominant Eukaryotic Organism among the Gut Contents

A total of 32 different phyla of eukaryotic organisms were identified from the gut contents of the three species of sea cucumbers. Of these, Diatomea was one of the main phyla in all the gut content samples but varied dramatically in relative abundance. Diatomea was the most abundant (41.61 ± 4.97%) in *S. monotuberculatus*. In *S. chloronotus*, Diatomea was also the most abundant (13.15 ± 1.48%) in the identified components, though its content was very close to phylum Apicomplexa (13.13 ± 4.77%). Moreover, phylum Diatomea was the second rich one (9.55 ± 2.36%) in the identified components of *H. atra*. In previous studies, Diatomea was found to be the predominant food source of some other holothurians [12,13,14,15,27,28]. A previous study also showed that diatom was the main food source for sea cucumber *Parastichopus parvimensis* [10]. Moreover, David et al. (2020) investigated the food sources and digestive efficiency of *H. forskali* based on the gut contents. They found the vegetal food sources ingested by *H. forskali* were mainly diatoms [46], which was similar to the results of the current research. Furthermore, we also found the content of *P. constrictum* was the highest among all diatom species in all samples of *S. monotuberculatus* (18.69–42.7%). Diatoms are commonly used diets for many aquaculture animals [47]; *P. constrictum* may be considered as an additive feed for captive breeding of *S. monotuberculatus*.

For *H. atra*, the most dominant phylum in the gut content samples was Apicomplexa (22.85 ± 7.33%), which was also the second richest one in *S. chloronotus* (13.13 ± 4.77%). Previous studies showed that Apicomplexa was ubiquitous in all major coral reef ecosystems, which was a core member of the coral microbiome [48]. In addition, some members of Apicomplexa were also found in the body cavities and intestines of invertebrates as parasites, and these parasites were transmitted commonly by the faecal–oral route path [49,50]. Because of that, we hypothesized that Apicomplexa species were introduced into sea cucumbers mainly through feeding process.

### 4.2. Comparison of Food Sources among Three Species of Sea Cucumbers

Although the eukaryotic dominant phylum with the largest proportion in the digestive tract of *H. atra* was different from that of the other two sea cucumber species, the β-diversity analysis and UPGMA clustering tree results showed that the eukaryotic food composition of *S. chloronotus* was much closer to that of *H. atra* than *S. monotuberculatus*. Previous studies have assessed the different habitat preferences of the three sea cucumber species. Bellchambers et al. (2011) found that *H. atra* was mainly distributed in the sandy bottom of the central lagoon, while *S. chloronotus* was widely distributed in the coral island area [51]. The three tropical sea cucumbers species in the South China Sea also had different habitat preferences: *H. atra* lived at the sandy bottom of the ocean and fed on coarse coral sand day and night; *S. chloronotus* was often exposed to the sand bottom of calm and lush seaweed; yet *S. monotuberculatus* lived under coral reefs or rocks and went out to feed at night [52]. In addition, the study of Eriksson et al. (2012) indicated *H. atra* was often found in all areas where *S. chloronotus* was found [53]. During the sample collection process in this study, *H. atra* and *S. chloronotus* were observed on the sandy bottom of a coral reef, while *S. monotuberculatus* was found under rocks (Figure 1). The habitats of sea cucumbers described by the above reports were consistent with the surrounding environments observed during sample collection in this study. Therefore, we inferred that the habitat preferences of *S. chloronotus* and *H. atra* were more accordant than that of *S. monotuberculatus*. Different habitats usually mean different food patches [54,55]. Ecological theory stated that niches of closely related species were usually separated, facilitating coexistence by reducing competition if food was limited [56]. The different habitat preferences of sea cucumbers meant that these closely related species occupied different ecological niches and may lead to obtaining different foods for reducing competition, which was consistent with the theory. The habitats of *H. atra* and *S. chloronotus* are more similar, so they bear a stronger resemblance of food composition. Likewise, as the habitats of the two are quite different from that of the sea cucumber *S. monotuberculatus*, the obvious distinction of food composition between them would be reasonable. That is, we conjectured that the eukaryotic food composition of *S. monotuberculatus* differed greatly from that of the other two sea cucumber species due to differences in habitat preferences.

Compared with *S. monotuberculatus*, *H. atra* and *S. chloronotus* had more similar intestinal eukaryotic microbial composition, but there were still differences. For example, the Apicomplexa content of *H. atra* was much higher than that of *S. chloronotus*, and the Diatomea content of the digestive tract in *H. atra* was lower than that of *S. chloronotus*. Previous studies indicated that compared with *H. atra*, *S. chloronotus* had a more certain feeding selectivity. The studies of Uthicke (1994, 1999) showed that *H. atra* fed on sediments with less microalgal biomass compared to *S. chloronotus*, and the latter species also selected sediment patches with finer particles than the former, which showed the different feeding strategies between two species [21,57]. Uthicke and Karez (1999) also found that *H. atra* exhibited no preference for any food type, which meant they did not deliberately choose to eat sediments with high microalgae concentrations. *S. chloronotus* distinctly selected sediments with the highest contents of microalgae and avoided the sediment with the lowest pigment concentrations [42], which also explained the differences in Diatomea content of gut among different species in this study. The holothurians use their tentacles to sweep or pick up surface sediments and feed on organic matter [58,59,60]. Previous research by the author’s group has shown there were different feeding selections among tropical sea cucumber species because of various tentacles [61,62,63]. For example, compared to *S. chloronotus* that generally feed on fine-grained sediments, the tentacle of *H. atra* is more suitable for feeding on coarser sediment particles [62]. There were obvious differences in organic matter among different sediments of particle sizes [64]. We speculate that the different compositions of prey between *H. atra* and *S. chloronotus* that live in similar habitats are possibly connected with the tentacles’ different morphology.

### 4.3. Relationship between Eukaryotic Communities in Guts and Sediments

In this study, the eukaryotic community composition among the gut content of three species of sea cucumbers and the surrounding sediments was compared, which indicated clearly different eukaryotic composition between the sediment and gut samples in *S. monotuberculatus*, *S. chloronotus* and *H. atra*. Compared with marine sediment samples, the contents of Annelida were almost nonexistent in samples of sea cucumbers, which were extremely high in the former (61.88 ± 16.59%). Multiple results of β-diversity, including NMDS analysis, PCoA analysis and UPGMA clustering tree, indicated that all the samples of three holothurians were obviously different compared to sediment samples. The results of α-diversity also suggested that surface sediments had a greater eukaryotic diversity than gut contents, but the guts of sea cucumbers had an enrichment effect on the number of some microorganisms. 

In detail, the relative contents of Diatomea, Arthropoda, Chlorophyta and Annelida in the gut of *S. monotuberculatus* were significantly different from those in sediments; the relative contents of Annelida in the gut of *S. chloronotus* were significantly different from those in sediments; the relative contents of Apicomplexa, Chlorophyta and Annelida in the gut of *H. atra* were significantly different from those in sediments. Some reports claimed that sea cucumbers feed selectively, particularly with respect to particle size, bacterial biomass, community composition and organic matter content of sediments [11,58,61,65,66,67,68,69]. Moreover, the structure of sea cucumber tentacles, consisting of size of nodules or nodule groups, inter-papillar spaces, mucous secretion ability of the nodules and sensory receptors at the terminal of each tentacle could be responsible for the physical and chemical selection for specific sediment patches [58,70,71,72]. For example, Foster and Hodgson (1996) found that different holothuroids species in the same intertidal area selected different sediments to feed because of differences in tentacle morphology. This selection strategy would probably certainly affect the feeding preference of sea cucumbers. Therefore, we speculate that the different eukaryotic communities in the gut contents and sediments may mainly result from selective feeding.

## 5. Conclusions

In this study, we compared the composition of eukaryotes in the guts of three different typical tropical sea cucumber species and surrounding sediments through the metabarcoding analysis of 18S rRNA gene V4 regions. Our study revealed that there were significant differences in eukaryotic composition either among three gut contents of sea cucumber or holothurians and sediments. We speculated that may be due to the feeding selectivity of sea cucumbers and the differences in tentacle morphology among different species of holothurians.

## Figures and Tables

**Table 1:** OTUs whose abundance exceeded 10% in single gut samples.

OTU	Phylum	Class	Species	Sample ID ^1^
OTU_1	Diatomea	Bacillariophyceae	*Psammodictyon constrictum*	Sm1
				Sm2
				Sm3
				Sm4
				Sm5
OTU_6	Arthropoda	Ostracoda	*Loxocorniculum mutsuense*	Sm1
				Sm5
OTU_185	Arthropoda	Ostracoda	*Loxocorniculum mutsuense*	Sm2
OTU_18	Mollusca	Gastropoda	Unidentified	Sm4
OTU_16	Arthropoda	Maxillopoda	Unidentified	Sm4
OTU_7	Apicomplexa	Gregarinasina	Unidentified	Sc1
				Ha3
				Ha5
OTU_27	Mollusca	Gastropoda	Unidentified	Sc1
OTU_4	Eukaryota	Unidentified	Unidentified	Sc2
				Sc5
				Ha1
				Ha3
OTU_15	Arthropoda	Maxillopoda	*Acartia pacifica*	Sc2
OTU_17	Eukaryota	Unidentified	Unidentified	Sc2
OTU_12	Chlorophyta	Unidentified	Unidentified	Sc4
OTU_8	Eukaryota	Unidentified	Unidentified	Ha2
OTU_10	Chromerida	Unidentified	*Chromerida* sp.RM11	Ha4
OTU_3219	Chromerida	Unidentified	*Chromerida* sp.RM11	Ha4
OTU_11	Apicomplexa	Gregarinasina	Unidentified	Ha5

^1^ Sm (S. monotuberculatus), Sc (S. chloronotus), Ha (H. atra).

## Data Availability

The datasets generated during this study have been uploaded to NCBI (No. PRJNA864035).

## Supplementary Materials

The following supporting information can be downloaded at: https://www.mdpi.com/article/10.3390/ani12172303/s1, Figure S1: Rarefaction Curve of the gut content and sediment samples. *S. monotuberculatus* (Sm), *S. chloronotus* (Sc) and *H. atra* (Ha); Table S1: OTUs whose abundance exceeded 1% but less than 10% in single gut samples.
