# Supplementary material for: Comparative Analysis of In Situ Eukaryotic Food Sources in Three Tropical Sea Cucumber Species by Metabarcoding
Source: Animals (Basel). 2022 Sep 5;12(17):2303. doi: 10.3390/ani12172303 (PMC9454777; doi:10.3390/ani12172303)
Supplement: Supplementary file 1 [file animals-12-02303-s001.zip › Table S1.pdf]

Table S1. OTUs whose abundance exceeded 1 % but less than 10 % in single gut samples.

OTU	Phylum	Class	Species	Sample ID[1]
OTU_2405	Arthropoda	Maxillopoda	*Acartia pacifica*	Sc3
OTU_2067	Annelida	Polychaeta	*Spio* sp. LK-2011-1	Sc3
OTU_72	Chlorophyta	Unidentified	*Chlorophyta* sp.CCMP1220	Sc4
OTU_37	Streptophyta	Unidentified	*Lactuca sativa*	Sc4
OTU_740	Phaeophyceae	Unidentified	*Lobophora variegata*	Sc5
OTU_530	Apicomplexa	Unidentified	*Gregarinasina* gen. sp. 4 KCW-2013	Sc5
OUT_2919	Diatomea	Bacillariophyceae	*Psammodictyon constrictum*	Sc1<br>Sc3<br>Sc4<br>Ha3<br>Ha4<br>Ha5
OTU_36	Chlorophyta	Ulvophyceae	*Parvocaulis parvulus*	Sc1<br>Ha3<br>Ha4
OTU_316	Eukaryota	Dinophyceae	*Symbiodinium* sp. Strain Kenting E	Sc5<br>Ha5
OTU_2238	Apicomplexa	Gregarinasina	*Gregarinidae* sp. from Phyllochaetopterus prolifica SR-2009a	Sc5<br>Ha2<br>Ha3<br>Ha5
OTU_1443	Eukaryota	Dinophyceae	*Symbiodinium* sp.	Sc1<br>Sc2<br>Sc3<br>Sc4<br>Sc5<br>Ha1<br>Ha2<br>Ha3<br>Ha5
OTU_73	Eukaryota	Unidentified	*Chromerida* sp. RM11	Sc1<br>Sc2<br>Sc4<br>Sc5<br>Ha2<br>Ha3<br>Ha5
OTU_1048	Eukaryota	Dinophyceae	*Symbiodinium* sp. Clade C	Sc1<br>Sc3<br>Sc4<br>Sc5<br>Ha1<br>Ha2<br>Ha4<br>Ha5
OTU_909	Chlorophyta	Unidentified	*Prasinophyte* sp. RCC856	Sc1

				Sc4<br>Ha1
OTU_2765	Eukaryota	Unidentified	*Karlodinium veneficum*	Ha3<br>Ha5
OTU_89	Apicomplexa	Gregarinasina	*Selenidium pendula*	Ha1
OTU_85	Arthropoda	Arachnida	*Rhombognathus levigatoides*	Ha5
OTU_790	Eukaryota	Dinophyceae	*Amphidinium* sp. FA1-CMSTAC022	Sm1<br>Sm2<br>Sm5<br>Ha3
OTU_1970	Phaeophyceae	Unidentified	*Sphacelaria* sp. UTEX LB 800	Sm3<br>Sc2<br>Sc5<br>Ha1
OTU_74	Eukaryota	Dinophyceae	*Amphidinium* sp. D1-CMSTAC020	Sm1<br>Sm5
OTU_102	Eukaryota	Dinophyceae	*Amphidinium* sp. FC2-CMSTAC023	Sm1
OTU_2568	Arthropoda	Ostracoda	*Hemicytherura kajiyamai*	Sm1<br>Sm3
OTU_75	Apicomplexa	Gregarinasina	*Lankesteria cystodytae*	Sm3
OTU_110	Mollusca	Gastropoda	*Clypeomorus brevis*	Sm3
OTU_2305	Arthropoda	Maxillopoda	*Scambicornus* sp. New Caledonia-RJH-2004	Sm3
OTU_6	Arthropoda	Ostracoda	*Loxocorniculum mutsuense*	Sm3

1: Sm (*S. monotuberculatus*), Sc (*S. chloronotus*), Ha (*H. atra*)
