# Supplementary figures and images for: Comparative Analysis of In Situ Eukaryotic Food Sources in Three Tropical Sea Cucumber Species by Metabarcoding
Source: Animals (Basel). 2022 Sep 5;12(17):2303. doi: 10.3390/ani12172303 (PMC9454777; doi:10.3390/ani12172303)

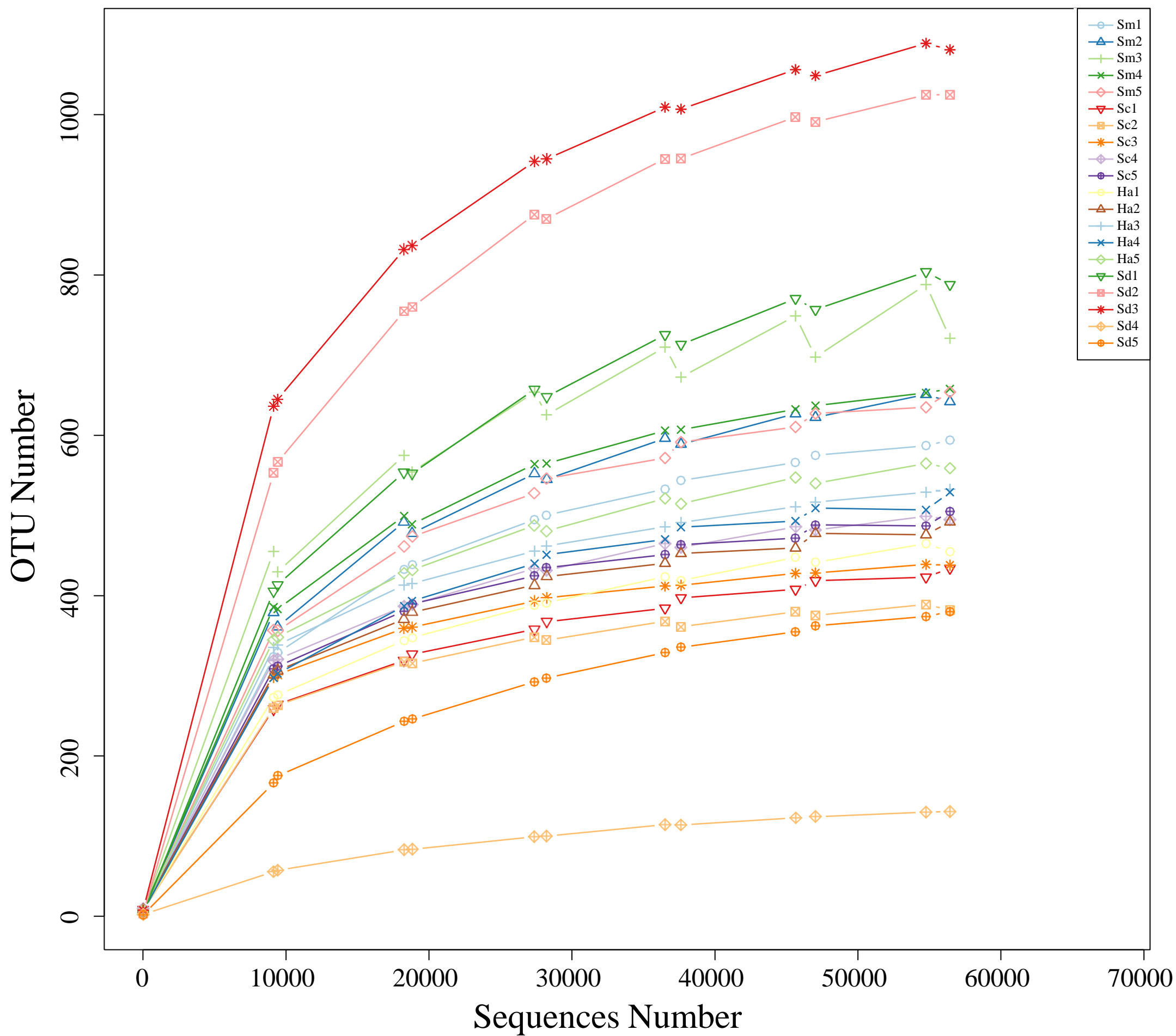

Supplement: Supplementary file 1 [file animals-12-02303-s001.zip › Figure S1.pdf]
